# Temporal trends in alcohol-related liver disease mortality and prior inpatient care in England before, during and after the COVID-19 pandemic (2004–2024): a population-based observational study

**DOI:** 10.1136/jech-2025-225179

**Published:** 2026-08-12

**Authors:** Emre Oguzman, Eva J A Morris, Matthew Quinn, Ian Rowe, Sasha Shepperd, James Webster, Xiaomin Zhong, Raph Goldacre

**Affiliations:** 1 Applied Health Research Unit, Nuffield Department of Population Health, University of Oxford, Oxford, UK; 2 Leeds Institute of Medical Research, University of Leeds, Leeds, UK; 3 Liver Unit, Leeds Teaching Hospitals NHS Trust, Leeds, UK

**Keywords:** COVID-19, MORTALITY, RECORD LINKAGE, ALCOHOLISM, LIVER CIRRHOSIS

## Abstract

**Background:**

Mortality from alcohol-related liver disease (ARLD) in England rose after the COVID-19 pandemic. Existing reports lack data on prior healthcare use, which is key to understanding care pathways before death. This study examined long-term trends in ARLD mortality before, during and after the pandemic and assessed changes in relation to prior inpatient care.

**Methods:**

In this population-based observational study using English national mortality records linked to NHS hospital data, all ARLD deaths in adults aged 25–84 were analysed from January 2004 to December 2024. Monthly mortality trends were examined by age, sex, deprivation, region and prior inpatient care. Interrupted time series estimated changes in mortality after the pandemic onset.

**Results:**

Over the study period, 97 058 ARLD deaths occurred, with about one-third out-of-hospital. Following the pandemic, ARLD mortality increased by 27.7% (95% CI 23.3% to 32.3%), representing 5571 excess deaths between March 2020 and December 2024. The step-increase was greater for out-of-hospital deaths (34.9%, 95% CI 28.4% to 41.7%) than for in-hospital deaths (22.9%, 95% CI 18.2% to 27.8%), and the increase in out-of-hospital deaths was driven by deaths in people who had no recent discharge in the previous year (75.7%, 95% CI 61.7% to 90.9%).

**Conclusions:**

The increase in deaths from ARLD has been driven by a disproportionate increase in those without recent inpatient care. Efforts to understand ARLD mortality should increasingly be focused on the out-of-hospital setting.

**WHAT IS ALREADY KNOWN ON THIS TOPIC**

- Deaths from alcohol-related liver disease (ARLD) are responsible for the rise in alcohol-specific mortality in England after the COVID-19 pandemic.
- Mortality outcomes for patients admitted to hospital with ARLD improved in the decade prior to the pandemic.
- ARLD has consistently been marked by missed opportunities for timely intervention, and the pandemic has disrupted access for managing alcohol misuse.

**WHAT THIS STUDY ADDS**

- Mortality from ARLD in England continues to remain high since the onset of the pandemic, with greater increases among those dying outside of hospital.
- The rise in out-of-hospital deaths from ARLD has been driven mainly by those with no recent inpatient care in the year preceding death, reflecting system and patient-level challenges in accessing support since the pandemic.

**HOW THIS STUDY MIGHT AFFECT RESEARCH, PRACTICE OR POLICY**

- Immediate large-scale public health measures are needed to respond to sustained elevated mortality from ARLD since the pandemic.
- Further research is needed to understand the care pathways of people dying of ARLD outside of hospital, with particular attention on those who die without any recent hospitalisation.

## Introduction

 Excessive alcohol consumption is a major risk factor for global mortality, accounting for over 1.8 million deaths and 56 million life years lost in 2021.^1^ Following the outbreak of the COVID-19 pandemic in March 2020, several countries in Europe and the USA saw increases in mortality from conditions wholly attributable to alcohol.^2 3^ Alcohol-related liver disease (ARLD) is an appropriate outcome for assessing mortality associated with population-level drinking patterns. In England, ARLD accounted for most alcohol-specific deaths between 2001 and 2022, and the increase in alcohol-specific mortality since 2019 has largely been driven by this condition.^4^ Before the pandemic, average weekly alcohol consumption was declining among adults in England.^5^ After its onset, high-risk drinking increased and has remained above prepandemic levels across sexes and socioeconomic groups,^6^ particularly among those who were heavier drinkers beforehand.^7^

The impact of these changes on ARLD mortality is captured by death registry from the Office for National Statistics (ONS).^8^ These data are subjected to delays of over a year and are aggregated annually, which limits detection of important episodic events and seasonal variation. Additionally, these data lack demographic detail, which is needed to identify populations at risk who would benefit most from targeted prevention and treatment strategies. If high-risk drinking patterns persist, more deprived populations are projected to experience the greatest burden of premature alcohol-related deaths.^9^

While outcomes after first emergency admissions for ARLD improved prior to the pandemic,^10^ it is unclear whether this trend has continued. Recent reports of increasing ARLD mortality omit information on place of death and prior healthcare utilisation, which would help distinguish recent from absent healthcare contact and identify potential missed opportunities for treatment. Such information may also help to inform where prevention strategies and policy change need to be targeted. Real-time monitoring is needed to track the growing burden of ARLD mortality and associated health inequalities to help inform policy interventions, but is absent.

The aim of this study was to investigate monthly ARLD mortality rates in England from 2004 to the present, overall and by recent inpatient care history and patient characteristics (age, sex, region of residence and area-level deprivation), and to quantify changes associated with the COVID-19 pandemic. Future updates via an interactive online dashboard will enable ongoing monitoring of ARLD mortality and inequalities across England.

## Methods

### Study design and data sources

This population-based observational study of adults aged 25–84 years used linked routinely collected health data. National mortality records from the ONS, supplied by NHS England, were used to identify all deaths in England between 1 January 2004 and 28 February 2025 where ARLD—International Classification of Diseases, 10th Revision (ICD-10) code K70—was recorded as the underlying cause of death.^11^ The K70 code has been previously validated with a positive predictive value of 98%.^12^ Deaths where ARLD was recorded as a contributing were also extracted for comparison. Mortality records were linked to Hospital Episode Statistics (HES) Admitted Patient Care records, collected and supplied by NHS England.^13 14^

Due to incomplete HES submissions,^15^ all records from NHS Frimley Integrated Care Board and NHS Shropshire, Telford and Wrekin Integrated Care Board were excluded to avoid underestimation of ARLD mortality, removing 1909 deaths (1.9% of national ARLD deaths). Further details are provided in the appendix (online supplemental etext 1).

Mortality records provided date of death occurrence and registration, age, sex, region of residence and area-level deprivation,^16^ with region and deprivation derived from the individual’s lower super output area using 2011 Census boundaries. From HES, admission and discharge dates, diagnoses and discharge method (including death in hospital) were extracted. Mid-year population estimates from ONS were used as denominators (online supplemental etext 1).

Temporal trends were assessed by age group (25–39, 40–54, 55–69 and 70–84 years), sex, region, deprivation (Index of Multiple Deprivation ranks grouped by quintiles), place of death and prior inpatient care in the year before death. Place of death and prior inpatient care were examined to distinguish deaths occurring with recent secondary care contact from those without recent hospital engagement. Inpatient episodes were categorised as those where ARLD (ICD-10 K70) was recorded as the main or other diagnosis code, and those with any diagnosis. The latter category was included to assess overall hospital service use in the year before death, irrespective of the reason for admission, and to identify recent hospital contact even when ARLD was not recorded.

Exact location of death outside hospital (eg, care home) is not available in the linked hospital mortality data. Previous literature indicates that most deaths from ARLD occur in hospital, followed by deaths at home, and a smaller proportion in other settings such as care homes.^17^ Deaths were considered to have occurred in-hospital if the patient had a discharge method code indicating death (*dismeth*=4) and out-of-hospital otherwise. Out-of-hospital deaths were grouped by time since last known ARLD-related hospital discharge: (1) within 60 days, (2) 61–365 days and (3) no ARLD-related discharge in the preceding 365 days. Trends in prior inpatient care for any reason during the same periods were assessed separately. To describe the population’s casemix and reasons for hospitalisation before death, the 20 most common admission diagnoses in the year prior to death were identified from any diagnosis position on the hospital record.

### Data completeness and study period

National mortality data are subjected to registration delays, which vary by age and cause of death,^18^ and are a particular problem for more recent data.^19^ To account for registration delays, we applied a correction factor to the final 9 months of the study based on empirical analysis of successive data cuts from ONS (online supplemental efigure 1). Given the substantial proportion of deaths that were deemed missing in the last 2 months, the analysis excluded the final 2 months of data received (January–February 2025). The final study period was January 2004 to December 2024, with the correction factor applied from April 2024 to December 2024.

The age range of the study population (25–84 years) accounted for 99.5% of all deaths from ARLD. Deaths were complete by region, deprivation and sex (>99.9%). Records with missing sex were excluded.

### Outcomes

The primary outcome was the monthly age-standardised population-based mortality rate (ASMR) where ARLD was the underlying cause of death.

### Statistical analyses

Mortality rates were computed using mid-year population estimates as denominators (online supplemental etext 1). Rates were age-standardised using the direct method with the 2013 European Standard Population as weights,^20^ expressed per million population. For each demographic characteristic (age group, sex, region and deprivation), a reference category was set and the mean annual crude rate was reported alongside the adjusted rate ratios, with 95% CIs based on a negative binomial distribution.

An interrupted time series design was used to quantify the temporal change in ARLD mortality after the onset of the COVID-19 pandemic. A step-change from March 2020 was hypothesised, reflecting an abrupt shift in population-level alcohol consumption and related harms rather than a gradual change in trend.^4^

Linear regression assuming a log-normal response was used to model the underlying monthly trend in the ASMR. A log-linear model allowed estimated effects to be interpreted as relative (percentage) changes and ensured positive fitted values. Time was included as a linear term, coded as successive monthly indices, to account for secular trends. The pandemic period was included as a binary term (0 before March 2020, 1 from March 2020 onwards) to estimate the immediate level change associated with the pandemic onset. Prepandemic rates were inspected visually to verify linearity. In sensitivity analysis, the prepandemic period was limited to January 2017–February 2020 to allow comparison of the postpandemic period with the more recent prepandemic period, irrespective of the longer term trend. Adjustment was made for seasonality using Fourier terms (two sine/cosine pairs over 12-month periods). This approach enabled smooth modelling of recurring seasonal patterns using relatively few parameters, in contrast to monthly indicator variables.^21^ The step-change coefficient and its 95% CI were obtained from the regression and expressed as a percentage: (exp(β)−1)×100. CIs were based on the Newey–West heteroskedasticity and autocorrelation consistent estimator with prewhitening and a lag length of 12 months. For descriptive purposes, the mean ASMR was calculated for the prepandemic and postpandemic periods from observed monthly rates, with CIs assuming a log-normal distribution.

Excess mortality from March 2020 was estimated as the difference between observed and expected numbers of deaths, reported monthly and cumulatively. Monthly death counts were modelled using negative binomial regression, with a log population offset, and adjusted for age, sex, time (in months) and seasonality. The model incorporated a categorical indicator for the pandemic period, specified as a single prepandemic reference category for all months before March 2020 and separate indicators for each subsequent month. Expected deaths were defined as the counterfactual number of deaths that would have been expected in the absence of the pandemic. Expected deaths for each pandemic month were obtained by dividing observed deaths by the corresponding estimated rate ratio for that month.

The overall trend analysis was repeated for deaths where ARLD appeared anywhere on the death certificate (underlying or contributing cause) to capture deaths where ARLD was secondary, for example, to COVID-19. As a sensitivity analysis to assess potential changes in outcome coding during the pandemic, the step-change from the interrupted time series model was re-estimated, excluding the peak pandemic period (March 2020–February 2021). To assess sensitivity to possible changes in death-registration workflows, the step-change was re-estimated by the analysis at three different postpandemic end dates: December 2021, December 2022 and December 2023.

Trends were assessed overall and by prior inpatient care and demographics. ARLD deaths with COVID-19 as a contributing cause were quantified and taken into account in all analyses.

R V.4.4.2 was used to conduct all statistical analyses.

## Results

Between January 2004 and December 2024, there were 97 058 deaths where ARLD was registered as the underlying cause among adults aged 25 to 84 years, with an average annual mortality burden of 128 deaths per million people. Mortality was largest in middle-aged adults, higher among the more deprived, and two times as high in men than in women. There was marked regional variation, with the North East and North West experiencing the highest standardised mortality rates (table 1; figure 1). Most ARLD deaths occurred in hospital (n=67 018, 69%) (table 1). Among out-of-hospital deaths (n=30 040, 31%), 56% (n=16 731) had no hospital-recorded ARLD diagnosis, and 36% (n=10 841) had no inpatient care in the year preceding death (online supplemental etable 1). After ARLD, the most common reason for hospital admission in the year before death was *Mental and behavioural disorders due to the use of alcohol* (ICD-10 F10; online supplemental etable 2), recorded in 76% of those diagnosed with ARLD and 34% of those without.

**Figure 1:**
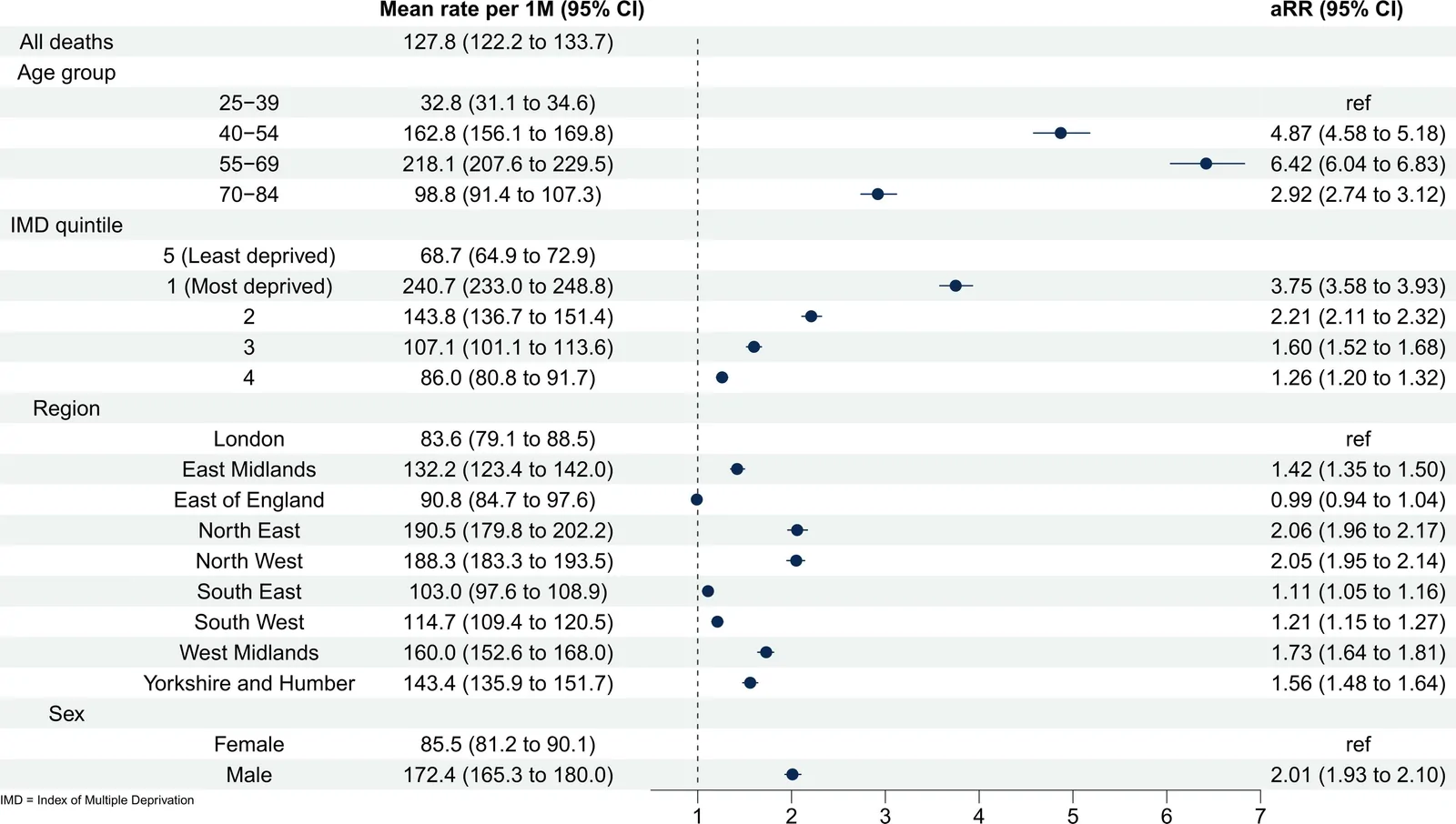
Alcohol-related liver disease (ICD-10 K70) deaths by demographics in England, January 2004 to September 2024: mean crude rates per million population per year and age-sex-adjusted rate ratios. ICD-10, International Classification of Diseases 10th Revision; IMD, Index of Multiple Deprivation.

**Table 1:** Distribution of deaths where alcohol-related liver disease (ICD-10 K70) was listed as an underlying cause in England between January 2004 and December 2024, by demographics, place of death and (among out-of-hospital deaths) presence of prior hospital-recorded diagnosis for ARLD

	In-hospital	ARLD discharge within 60d	ARLD discharge within 61 days–1 year	No ARLD discharge within 1 year	Out-of-hospital	Total
All deaths	67 018 (69)	8149 (8.4)	5160 (5.3)	16 731 (17.2)	30 040 (31)	97 058 (100)
Age group						
25–39	4853 (64.8)	614 (8.2)	462 (6.2)	1559 (20.8)	2635 (35.2)	7488 (100)
40–54	24 805 (67.2)	2850 (7.7)	2289 (6.2)	6993 (18.9)	12 132 (32.8)	36 937 (100)
55–69	29 247 (71)	3361 (8.2)	1919 (4.7)	6677 (16.2)	11 957 (29)	41 204 (100)
70–84	8113 (71)	1324 (11.6)	490 (4.3)	1502 (13.1)	3316 (29)	11 429 (100)
Sex						
Female	24 192 (72.6)	2461 (7.4)	1617 (4.8)	5071 (15.2)	9149 (27.4)	33 341 (100)
Male	42 826 (67.2)	5688 (8.9)	3543 (5.6)	11 660 (18.3)	20 891 (32.8)	63 717 (100)
IMD quintile						
1 (most deprived)	23 645 (68.5)	2774 (8)	1858 (5.4)	6221 (18)	10 853 (31.5)	34 498 (100)
2	15 144 (68.9)	1826 (8.3)	1188 (5.4)	3811 (17.3)	6825 (31.1)	21 969 (100)
3	11 538 (68.9)	1454 (8.7)	868 (5.2)	2892 (17.3)	5214 (31.1)	16 752 (100)
4	9388 (69.8)	1140 (8.5)	743 (5.5)	2176 (16.2)	4059 (30.2)	13 447 (100)
5 (least deprived)	7303 (70.3)	955 (9.2)	503 (4.8)	1631 (15.7)	3089 (29.7)	10 392 (100)
Region						
East Midlands	6232 (70.3)	743 (8.4)	465 (5.2)	1422 (16)	2630 (29.7)	8862 (100)
East of England	5371 (67.7)	673 (8.5)	409 (5.2)	1482 (18.7)	2564 (32.3)	7935 (100)
London	6748 (67.9)	677 (6.8)	484 (4.9)	2024 (20.4)	3185 (32.1)	9933 (100)
North East	5198 (72.3)	617 (8.6)	330 (4.6)	1047 (14.6)	1994 (27.7)	7192 (100)
North West	13 248 (68.8)	1544 (8)	1044 (5.4)	3425 (17.8)	6013 (31.2)	19 261 (100)
South East	8212 (67.9)	1081 (8.9)	647 (5.3)	2154 (17.8)	3882 (32.1)	12 094 (100)
South West	6217 (68.4)	808 (8.9)	611 (6.7)	1449 (15.9)	2868 (31.6)	9085 (100)
West Midlands	8106 (68.6)	1023 (8.7)	604 (5.1)	2077 (17.6)	3704 (31.4)	11 810 (100)
Yorkshire and the Humber	7686 (70.6)	983 (9)	566 (5.2)	1651 (15.2)	3200 (29.4)	10 886 (100)

Columns 3–5 are subcategories of out-of-hospital deaths (column 6); columns 2 and 6 sum to the total in column 7.

ARLD, alcohol-related liver disease; ICD-10, International Classification of Diseases 10th Revision; IMD, Index of Multiple Deprivation.

Figure 2 presents the time series of the ASMR between January 2004 and December 2024. From 2004 to 2019, the mean monthly ASMR was stable at around 10 deaths per million. Following the onset of the COVID-19 pandemic (March 2020 onwards), rates increased significantly and remained elevated, with an overall step-increase of 27.7% (95% CI 23.3% to 32.3%). The step-increase was greater for out-of-hospital deaths (34.9%, 95% CI 28.4% to 41.7%) than for in-hospital deaths (22.9%, 95% CI 18.2% to 27.8%) (figure 2). Among out-of-hospital deaths, the largest increase was observed in those without an ARLD-related hospital discharge in the year before death (61.5%, 95% CI 51.0% to 72.8%) (figure 3).

**Figure 2:**
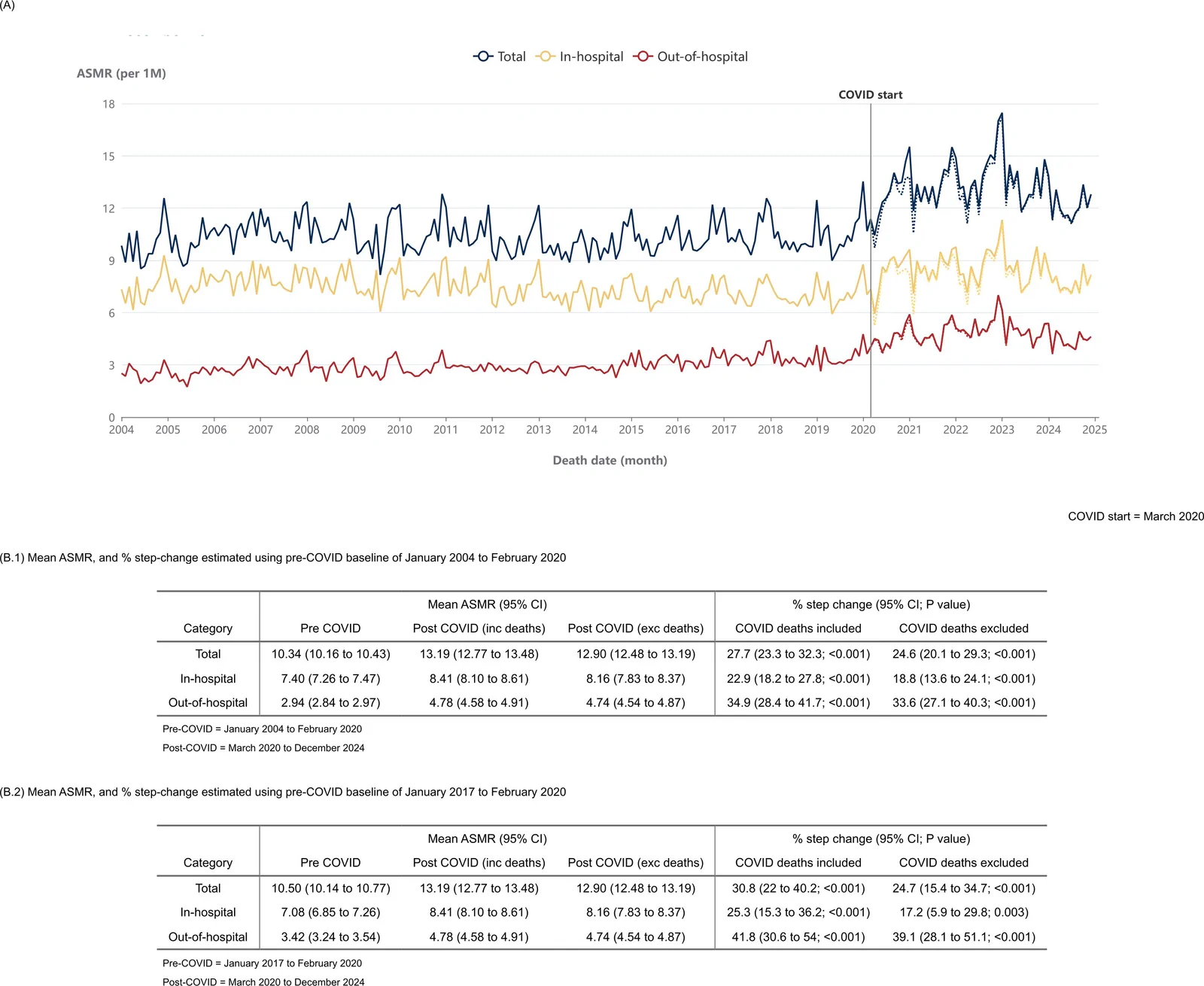
Time series of alcohol-related liver disease (ICD-10 K70) mortality in England by place of death, January 2004–December 2024. ASMR, age-standardised mortality rates; ICD-10, International Classification of Diseases 10th Revision.

**Figure 3:**
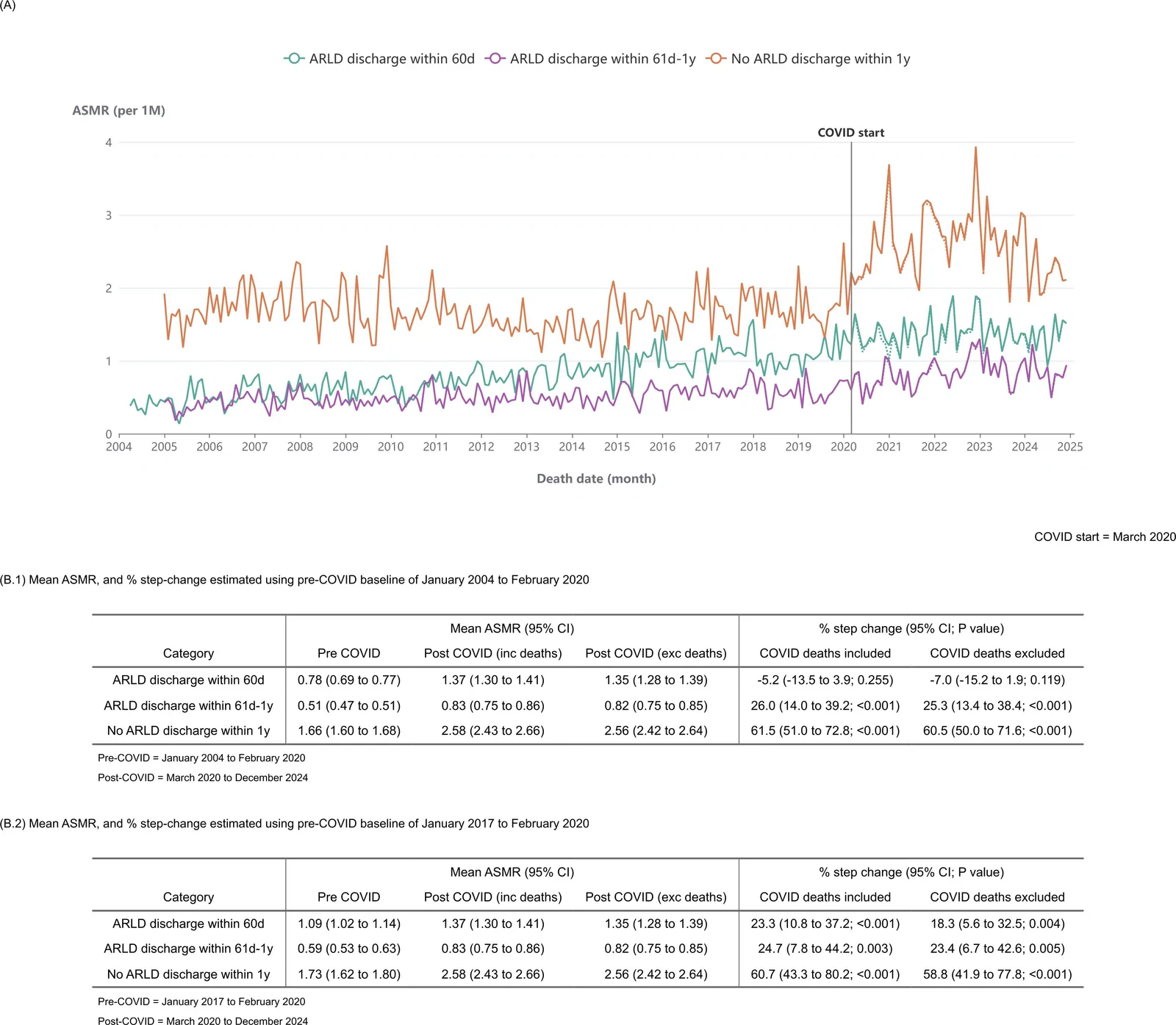
Time series of alcohol-related liver disease (ICD-10 K70) mortality in individuals who died out-of-hospital in England, by presence of recent hospital discharge with ARLD, January 2004–December 2024. ARLD, alcohol-related liver disease; ASMR, age-standardised mortality rates; ICD-10, International Classification of Diseases 10th Revision.

Restricting the prepandemic period to January 2017–February 2020 did not substantively alter the observed step-increases when split by place of death (figure 2), but it resulted in a step-increase in out-of-hospital deaths where ARLD was recorded within the previous 60 days (23.3%, 95% CI 10.8% to 37.2%), highlighting the importance of accounting for long-term trends since 2004 (figure 3). Individuals with no inpatient care of any kind experienced a larger postpandemic increase in ARLD mortality (75.7%–95% CI 61.7% to 90.9%) (online supplemental efigure 2).

The step-increase in ASMRs corresponded to a cumulative excess of 5571 ARLD deaths between March 2020 and December 2024, including 2315 out-of-hospital and 3256 in-hospital deaths (online supplemental efigure 3). Excess deaths were concentrated among individuals without prior ARLD-related hospital discharge (1952 deaths) (online supplemental efigure 4), and among those with no inpatient care the preceding year (1360 deaths) (online supplemental efigure 5).

Postpandemic mortality continued to reflect long-standing inequalities, remaining higher among men, middle-aged adults, residents of more deprived areas and those living in the North East and North West (figure 4). These differences persisted when stratified by place of death (online supplemental efigure 6). All demographic subgroups experienced similar step-increases after March 2020 (figure 4), although regional variation was evident when split by place of death (online supplemental efigure 6). For example, out-of-hospital deaths increased by 56.7% (95% CI 39.5% to 76.1%) in the West Midlands, while there was no clear increase in the North East (9.5%, 95% CI −6.9% to 28.7%).

**Figure 4:**
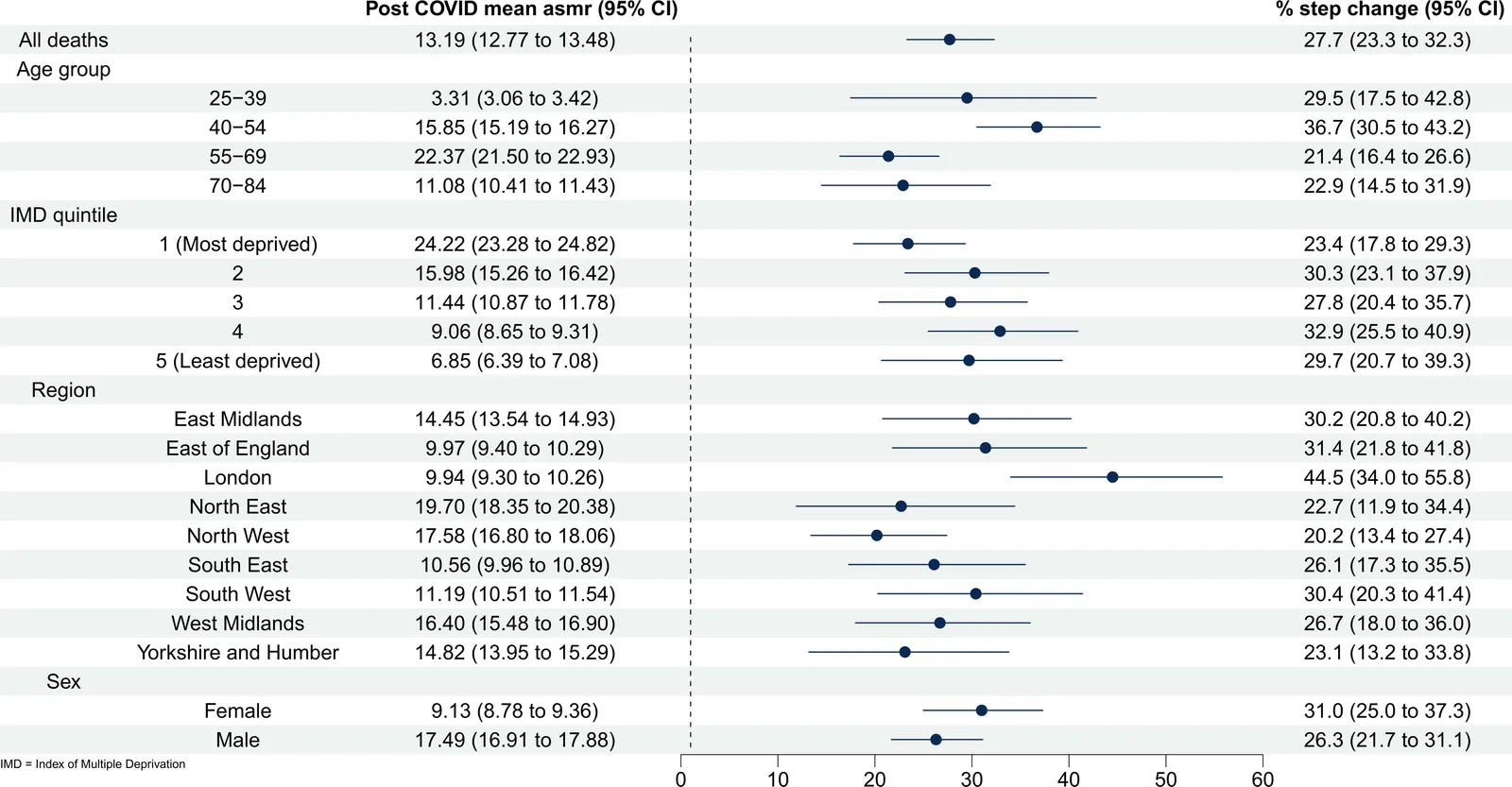
COVID-19-associated percentage change in age-standardised mortality rates (ASMR) for alcohol-related liver disease (ICD-10 K70) in England, by demographics. ICD-10, International Classification of Diseases 10th Revision; IMD, Index of Multiple Deprivation.

Deaths mentioning ARLD anywhere on the death certificate were 29% higher than those where ARLD was the underlying cause, a margin that remained stable over time (online supplemental figure 7a). The overall step-increase for deaths mentioning ARLD anywhere was 27.0% (95% CI 22.8% to 31.3%) (online supplemental figure 7b), corresponding to 7186 excess deaths between March 2020 and December 2024.

Results were robust to all sensitivity analyses, including exclusion of the peak pandemic period and variation of the postpandemic end date.

## Discussion

ARLD mortality in England rose 28% above expected levels after March 2020 and remained elevated through 2024. The largest increases were in individuals who died outside hospital and had no recent hospital-recorded ARLD diagnosis; these individuals comprised 35% of all postpandemic ARLD deaths. All demographic subgroups experienced increases in ARLD mortality after March 2020 that varied by prior hospital care.

This population-based study provides the most contemporary information on ARLD mortality in England since the pandemic and is the first to present prepandemic and postpandemic trends by prior inpatient care. It extends earlier work showing postpandemic increases in ARLD mortality in England, overall and by age, sex and deprivation, up to 2022,^4^ and adds updated evidence on place of death.^22^ Similar increases have been reported in Europe and the USA,^2 23^ but more research is needed to examine ARLD mortality by prior inpatient care in other contexts. Examining deaths by prior inpatient care is important for identifying missed opportunities for earlier recognition and intervention, which were noted for ARLD prior to the pandemic.^10 24^

Given the long latency of ARLD, most individuals who died in the postpandemic period developed harmful alcohol use years earlier. For some, acute-on-chronic liver failure may have worsened from increased, high-risk drinking during the pandemic.^7 25^ Projections of excess mortality in England have recently been modelled under various scenarios of alcohol consumption during and after the pandemic.^7 9^ Direct comparison of these projections with the excesses observed in the present study is not possible, as the projections considered mortality for a range of different alcohol-related conditions and amount of alcohol consumed. However, ARLD remains the largest contributor to wholly alcohol-related mortality, both before and after the pandemic.^4^ In the present study, excluding COVID-19 deaths, approximately 5000 excess ARLD deaths occurred between March 2020 and December 2024, rising to over 7000 when including deaths where ARLD was recorded as a contributing cause. These findings are consistent with evidence that heavier drinkers increased consumption during the pandemic and did not subsequently reduce intake.^7^

The postpandemic increase in deaths without any recent hospitalisation, for ARLD or otherwise, may reflect system and patient-level challenges in accessing support. Pandemic-related lockdown restrictions disrupted health systems globally, and ARLD treatment and management were both affected.^26 27^ In England, this occurred alongside reduced funding for specialist services and increased demand for alcohol misuse support.^28 29^ Service delivery has also transformed, with telemedicine becoming a widespread substitute for in-person treatment.^27^ Although remote service provision improved access for some, it may have excluded individuals with limited digital access or complex social and mental health needs, but these factors are not captured in the linked mortality-hospitalisation data used here.^30 31^

Pre-existing inequalities in ARLD mortality have persisted since the pandemic,^4^ with the largest absolute increases occurring among men, middle-aged adults and those from the most deprived areas. Novel disparities emerged when stratifying by place of death: women, those residing in the North West or West Midlands, and those from the most deprived areas all experienced a greater relative increase in out-of-hospital than in-hospital deaths. All-cause mortality among patients diagnosed with ARLD in primary care disproportionately affected women and the most deprived groups before the pandemic.^32^ Studies from the USA have reported pandemic-related inequities in sex, including greater loneliness, stress and psychological distress among women than men, all linked to greater alcohol consumption.^27^ The increase in out-of-hospital mortality for the West Midlands is concerning: in 2022, it reportedly had the highest proportion of non-drinkers and the lowest estimated maximum alcohol consumption of any English region.^33^ Mortality in these groups will continue to be monitored as policy evolves.^34^

Future research should focus on the extent of health service engagement beyond inpatient care, the profile of people who died without recent hospitalisation since the pandemic, and regional differences, including the increase in out-of-hospital mortality observed in the West Midlands. It is essential to understand whether patterns of primary care and other hospital service use, including outpatient and accident and emergency, have changed among decedents since the pandemic. Identification of high-risk drinking and intervention at the point of primary care is critical in preventing end-stage disease.^35^

The overall and sustained step-increase in ARLD mortality underscores an immediate need to consider broad public health strategies to mitigate alcohol-related harm, such as stricter alcohol marketing regulations and minimum unit pricing.^36^ The Institute of Alcohol Studies has expressed concern that the UK government’s recent 10-Year Health Plan fails to include such policies.^36 37^ Prevention measures are essential, given the current lack of specific medical treatments for decompensated ARLD.^38^ An online dashboard has been developed to provide near real-time monitoring of mortality rates at the monthly level, which provides greater temporal detail and speed of publication than other dashboards available from the ONS or the Office for Health Improvement and Disparities.^8 34 39^

### Strengths and limitations

This is the first study internationally of long-term trends in alcohol-related mortality before, during and after the pandemic, stratified by prior inpatient care and place of death. Linkage of national multiple-cause coded mortality records with two decades of HES records enabled identification of all causes of death and hospital admission among ARLD decedents, and control for the direct effect of COVID-19 infection. The close tracking of trends in which ARLD was recorded as the underlying cause, compared with cases where ARLD appeared anywhere on the death certificate, avoided potential variation in recording ARLD. The availability of up to 20 diagnoses on each hospital record meant that ARLD diagnosed in hospital could be ascertained with reference to the primary or any other diagnosis. This approach is useful as the National Confidential Enquiry into Patient Outcome and Death survey revealed variation in the total number of patients admitted with a primary diagnosis for ARLD due to differences in coding of primary diagnosis between different hospitals.^40^

Limitations include the lack of data on the use of other services, including primary care and specialised treatment, which may prevent or treat alcohol misuse. A prepandemic study on first emergency admissions for ARLD using linked CPRD and HES records revealed a low proportion of liver disease being recorded among patients with primary care visits in the year before admission.^10^ It is unclear whether this proportion has changed in the aftermath of the pandemic. Registration timeliness may have also changed differently across age groups due to shifts in healthcare utilisation during the pandemic.^41^ Studies from the USA have shown marked differences in ARLD outcomes by ethnicity^27^; however, ethnicity was not available for this study. Finally, incomplete HES submissions from two NHS trusts required the exclusion of two ICBs, which may have introduced a small risk of selection bias; however, these areas accounted for a limited proportion of national ARLD deaths.

## Conclusions

The persistent elevation of ARLD mortality rates since the pandemic, observed across all demographic groups, represents the tip of the iceberg for high-risk drinking behaviours and underscores an immediate need for broad public health strategies to mitigate alcohol-related harm. The disproportionately high increase in out-of-hospital ARLD deaths likely reflects system-level and patient-level challenges in accessing support since the pandemic, highlighting a need to diversify strategies for identifying individuals with established alcohol misuse. Further research using linked primary care data is needed to better understand healthcare utilisation patterns and the drivers of increased out-of-hospital ARLD deaths. Mortality trends by prior inpatient care and demographic groups will continue to be monitored via an online dashboard to track progress.^34^

## Supplementary material

10.1136/jech-2025-225179 online supplemental figure 1

10.1136/jech-2025-225179 online supplemental figure 2

10.1136/jech-2025-225179 online supplemental figure 3

10.1136/jech-2025-225179 online supplemental figure 4

10.1136/jech-2025-225179 online supplemental figure 5

10.1136/jech-2025-225179 online supplemental figure 6

10.1136/jech-2025-225179 online supplemental figure 7

10.1136/jech-2025-225179 online supplemental table 1

10.1136/jech-2025-225179 online supplemental table 2

10.1136/jech-2025-225179 online supplemental file 1

## Data Availability

Data may be obtained from a third party and are not publicly available.
