# Supplementary material for: Temporal trends in alcohol-related liver disease mortality and prior inpatient care in England before, during and after the COVID-19 pandemic (2004–2024): a population-based observational study
Source: J Epidemiol Community Health. 2026 Aug 12;80(11):e225179. doi: 10.1136/jech-2025-225179 (PMC13691703; doi:10.1136/jech-2025-225179)
Supplement: Supplementary file 1 [file jech-80-11-s001.pdf]

eFigure 1. Time series of estimated completeness of receipt of death certifications where alcohol-related liver disease (ICD-10 K70) was listed as an underlying cause in England, by age group.

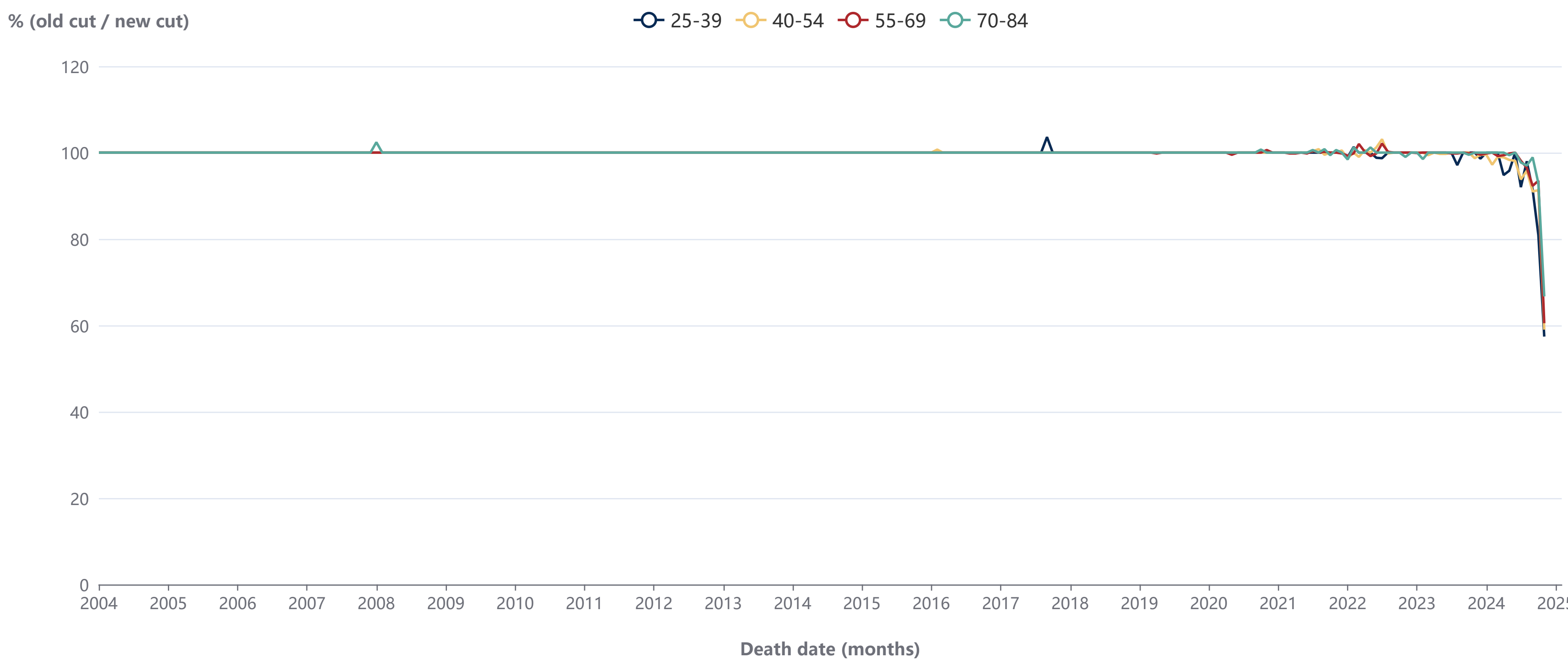

Footnote: Completeness was estimated from comparison of death counts in an “older” data extract (ending November 2024) with a “newer” extract (ending February 2025). This comparison revealed the extent to which, for each month of the study period, the counts in the earlier extract fall short of the counts in the later extract. Based on these observed differences, we applied an uplift to the last nine months of data in the latest extract to obtain corrected numbers by applying the following calculation: Obs/(C%), where Obs is the number of hospital admissions observed in each final month of the latest data extract and C% is that month’s estimated completeness based on the comparison of successive extracts.
