# Supplementary material for: Temporal trends in alcohol-related liver disease mortality and prior inpatient care in England before, during and after the COVID-19 pandemic (2004–2024): a population-based observational study
Source: J Epidemiol Community Health. 2026 Aug 12;80(11):e225179. doi: 10.1136/jech-2025-225179 (PMC13691703; doi:10.1136/jech-2025-225179)

eFigure 3. Time series of post-COVID excess deaths from alcohol-related liver disease (ICD-10 K70) in England by place of death, March 2020 to December 2024. Monthly (A) difference and (B) cumulative difference between observed and predicted counts of deaths, including and excluding where COVID-19 (U07.1, U07.2) was listed on death certificate. Excess deaths adjusted for population size, age, sex, underlying temporal trend (in months), and seasonality.

(A)

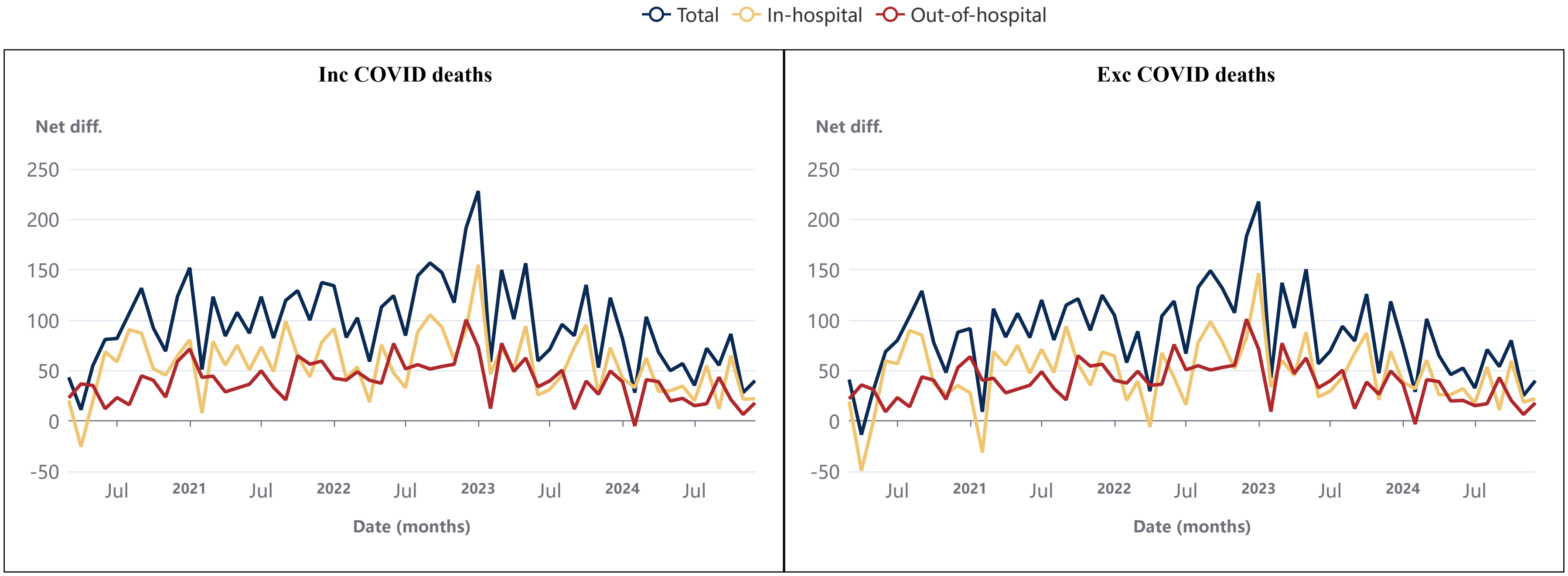

(B)

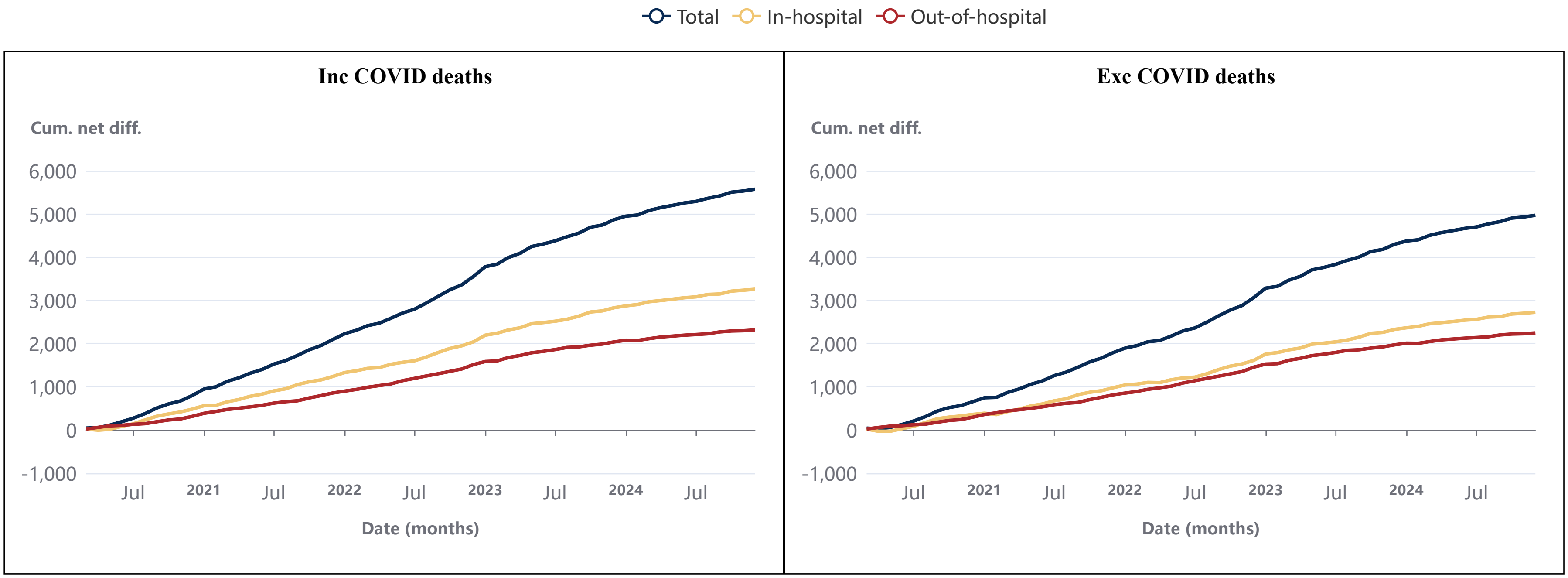

Supplement: Supplementary file 3 [file jech-80-11-s003.pdf]
