# Supplementary material for: Temporal trends in alcohol-related liver disease mortality and prior inpatient care in England before, during and after the COVID-19 pandemic (2004–2024): a population-based observational study
Source: J Epidemiol Community Health. 2026 Aug 12;80(11):e225179. doi: 10.1136/jech-2025-225179 (PMC13691703; doi:10.1136/jech-2025-225179)

eFigure 5. Time series of post-COVID excess deaths from alcohol-related liver disease (ICD-10 K70) in individuals who died out-of-hospital in England by presence of any recent hospital discharge, March 2020 to December 2024. Monthly (A) difference and (B) cumulative difference between observed and predicted counts of deaths, including and excluding where COVID-19 (U07.1, U07.2) was listed on death certificate. Excess deaths adjusted for population size, age, sex, underlying temporal trend (in months), and seasonality.

(A)

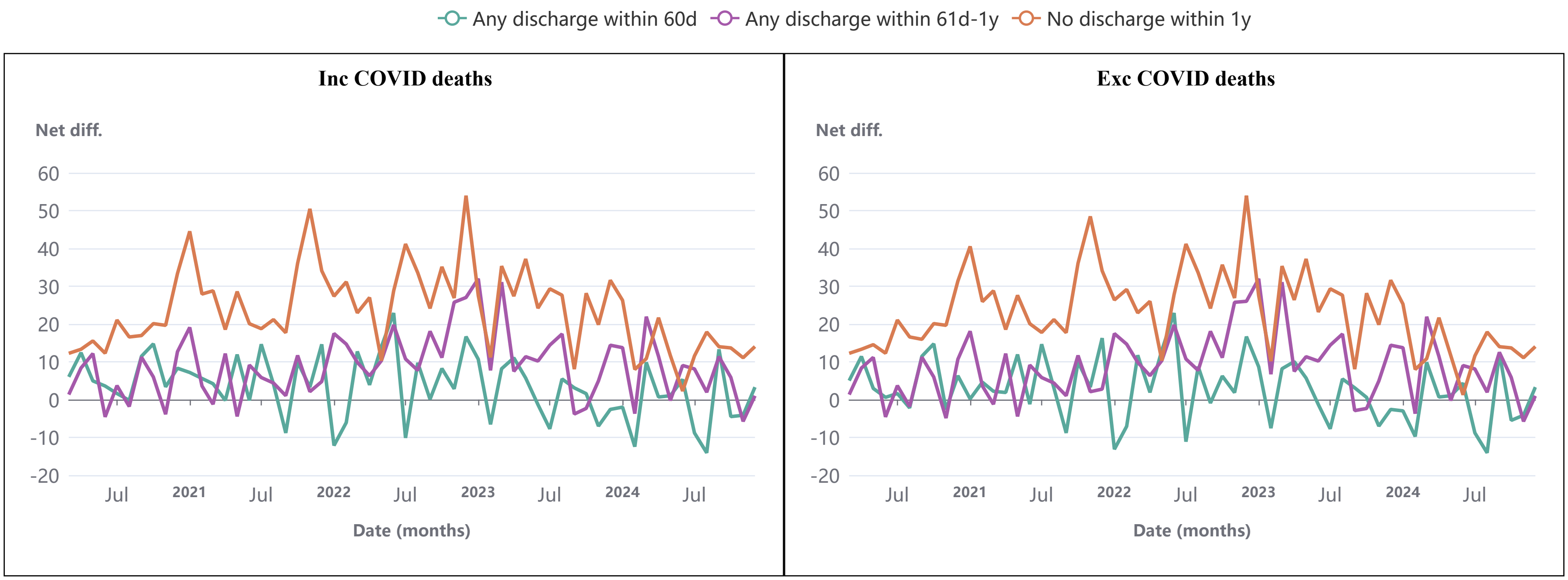

(B)

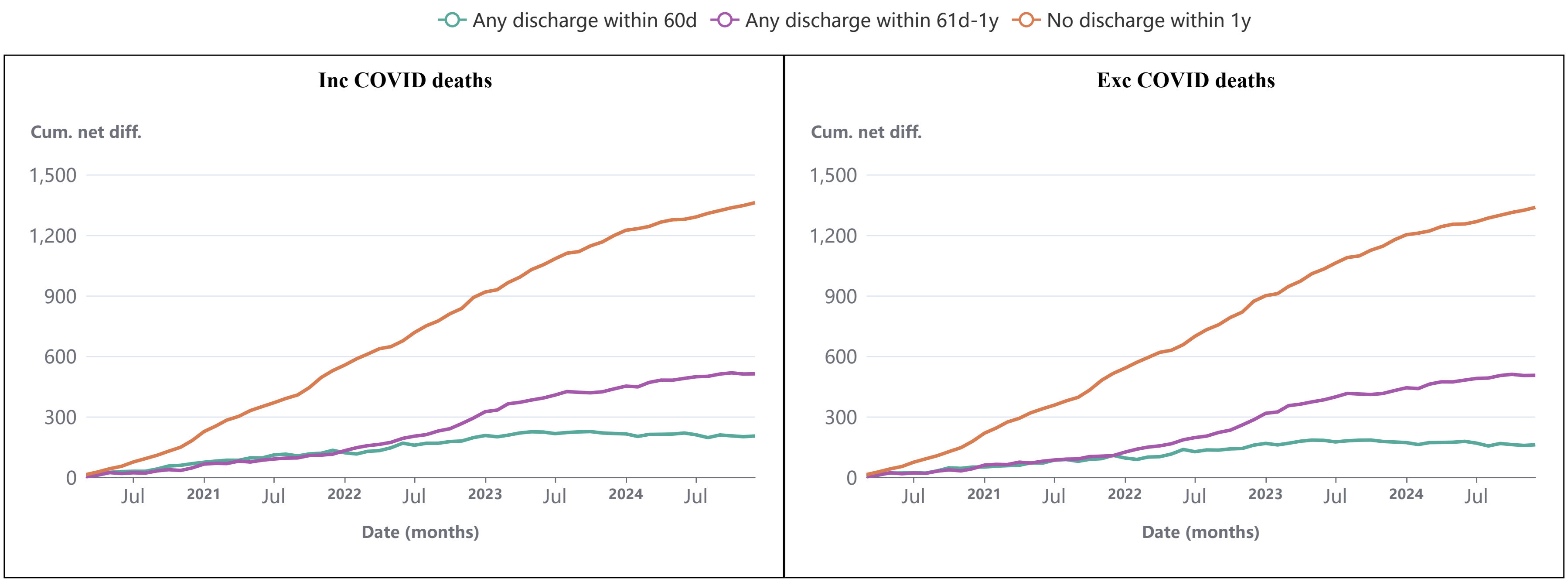

Supplement: Supplementary file 5 [file jech-80-11-s005.pdf]
