# Supplementary material for: Temporal trends in alcohol-related liver disease mortality and prior inpatient care in England before, during and after the COVID-19 pandemic (2004–2024): a population-based observational study
Source: J Epidemiol Community Health. 2026 Aug 12;80(11):e225179. doi: 10.1136/jech-2025-225179 (PMC13691703; doi:10.1136/jech-2025-225179)
Supplement: Supplementary file 6 [file jech-80-11-s006.pdf]

eFigure 6. COVID−19−associated percentage change in age−standardised mortality rates (ASMR) for alcohol−related liver disease (ICD−10 K70) in England, by demographics, stratified by place of death. Percentage changes adjusted for underlying temporal trend (in months) and seasonality.

	Post COVID mean ASMR (95% CI)	% step change (95% CI)
All deaths	8.41 (8.10 to 8.61)	22.9 (18.2 to 27.8)
	4.78 (4.58 to 4.91)	34.9 (28.4 to 41.7)
Age group		
25−39	2.04 (1.84 to 2.11)	20.3 (7.9 to 34.1)
	1.47 (1.30 to 1.52)	37.2 (16.7 to 61.4)
40−54	9.83 (9.30 to 10.13)	33.0 (26.3 to 40.0)
	6.01 (5.66 to 6.20)	41.7 (31.1 to 53.2)
55−69	14.68 (14.06 to 15.06)	16.1 (10.6 to 22.0)
	7.69 (7.24 to 7.93)	30.7 (22.6 to 39.3)
70−84	7.11 (6.57 to 7.35)	20.1 (10.6 to 30.4)
	4.01 (3.69 to 4.15)	16.4 (2.9 to 31.6)
IMD quintile		
1 (Most deprived)	15.17 (14.53 to 15.57)	17.0 (11.2 to 23.1)
	9.04 (8.48 to 9.33)	34.5 (24.4 to 45.5)
2	10.12 (9.58 to 10.42)	24.5 (16.7 to 32.8)
	5.86 (5.48 to 6.05)	41.5 (28.6 to 55.6)
3	7.34 (6.88 to 7.58)	25.2 (16.3 to 34.9)
	4.09 (3.75 to 4.24)	30.4 (17.6 to 44.7)
4	5.91 (5.64 to 6.07)	29.4 (20.7 to 38.8)
	3.14 (2.79 to 3.26)	36.7 (20.9 to 54.5)
5 (Least deprived)	4.49 (4.13 to 4.65)	26.3 (16.6 to 36.8)
	2.36 (2.14 to 2.44)	37.8 (19.1 to 59.5)
Region		
East Midlands	9.37 (8.70 to 9.69)	26.9 (16.8 to 37.9)
	5.08 (4.44 to 5.27)	36.2 (16.9 to 58.6)
East of England	6.34 (5.95 to 6.55)	31.7 (20.5 to 43.9)
	3.63 (3.11 to 3.78)	23.4 (4.9 to 45.0)
London	6.21 (5.72 to 6.43)	41.3 (28.6 to 55.3)
	3.72 (3.25 to 3.86)	49.5 (27.4 to 75.3)
North East	14.06 (12.87 to 14.58)	28.2 (15.2 to 42.6)
	5.63 (4.90 to 5.84)	9.5 (−6.9 to 28.7)
North West	10.64 (10.08 to 10.95)	8.6 (1.8 to 15.9)
	6.92 (6.48 to 7.15)	42.6 (30.2 to 56.1)
South East	6.76 (6.29 to 6.99)	25.5 (15.8 to 36.1)
	3.80 (3.35 to 3.94)	23.2 (9.2 to 39.0)
South West	7.06 (6.47 to 7.31)	26.8 (15.7 to 39.1)
	4.12 (3.65 to 4.27)	33.8 (16.1 to 54.2)
West Midlands	10.10 (9.38 to 10.44)	13.0 (3.8 to 23.1)
	6.29 (5.77 to 6.50)	56.7 (39.5 to 76.1)
Yorkshire and Humber	9.82 (9.18 to 10.16)	24.4 (13.6 to 36.2)
	5.00 (4.46 to 5.18)	16.7 (2.3 to 33.1)
Sex		
Female	6.21 (5.93 to 6.38)	24.9 (18.4 to 31.7)
	2.92 (2.77 to 3.00)	45.4 (35.0 to 56.6)
Male	10.73 (10.33 to 10.99)	21.8 (16.9 to 27.0)
	6.75 (6.43 to 6.94)	31.1 (24.3 to 38.2)

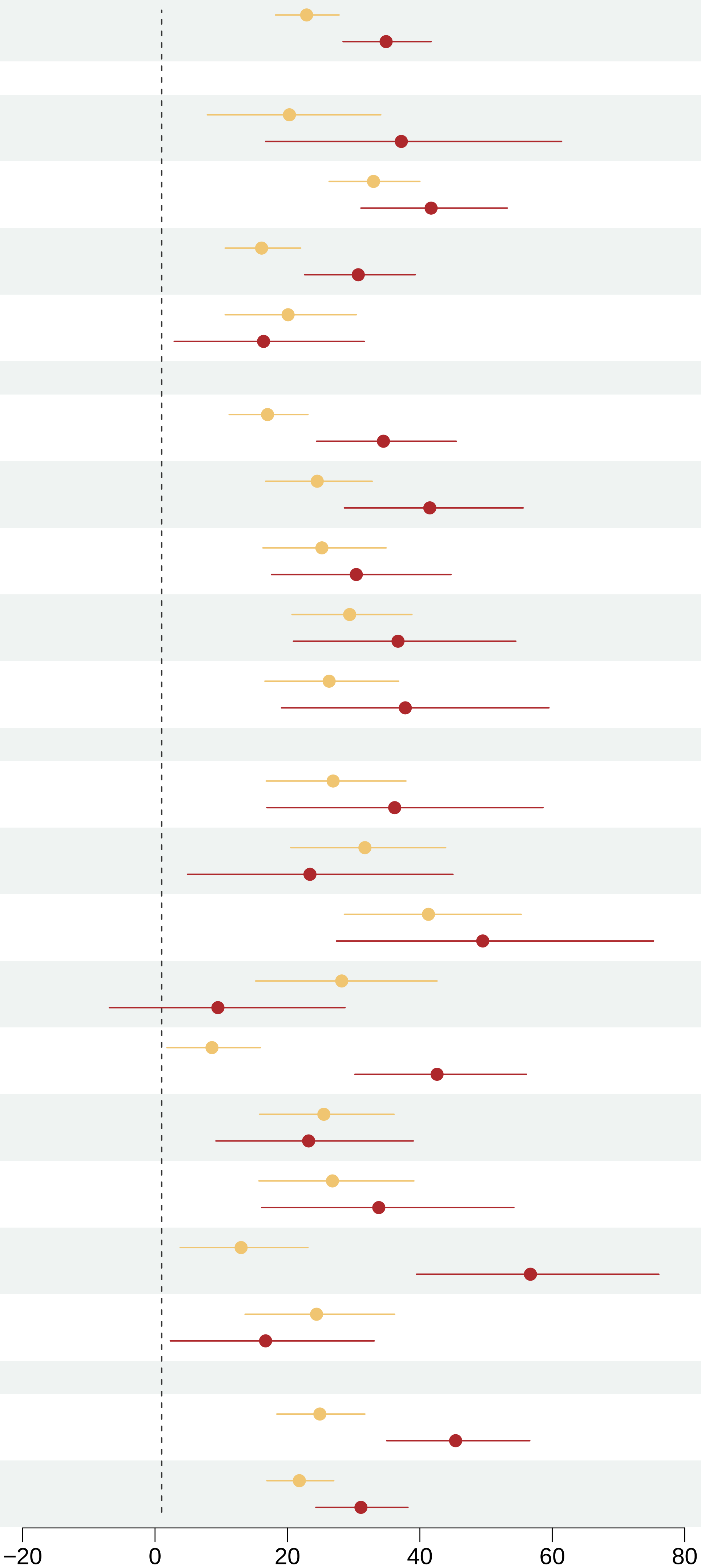

Death location
In−hospital
Out−of−hospital

IMD = Index of Multiple Deprivation
