# Supplementary material for: Temporal trends in alcohol-related liver disease mortality and prior inpatient care in England before, during and after the COVID-19 pandemic (2004–2024): a population-based observational study
Source: J Epidemiol Community Health. 2026 Aug 12;80(11):e225179. doi: 10.1136/jech-2025-225179 (PMC13691703; doi:10.1136/jech-2025-225179)
Supplement: Supplementary file 7 [file jech-80-11-s007.pdf]

eFigure 7. Time series of alcohol-related liver disease (ICD-10 K70) mortality in England by cause of death classification, January 2004-December 2024. A. Plot of monthly age-standardised mortality rates (ASMR). B1. Pre- and post-pandemic mean ASMR, and pandemic-associated percentage change, relative to the long-term pre-pandemic trend (since 2004). B2. Pre- and post-pandemic mean ASMR, and pandemic-associated percentage change, relative to the short-term pre-pandemic trend (since 2017). Rates expressed per 1,000,000, including (solid line) and excluding (dotted line) where COVID-19 (U07.1, U07.2) was listed on death certificate. Percentage changes adjusted for underlying temporal trend (in months) and seasonality.

(A)

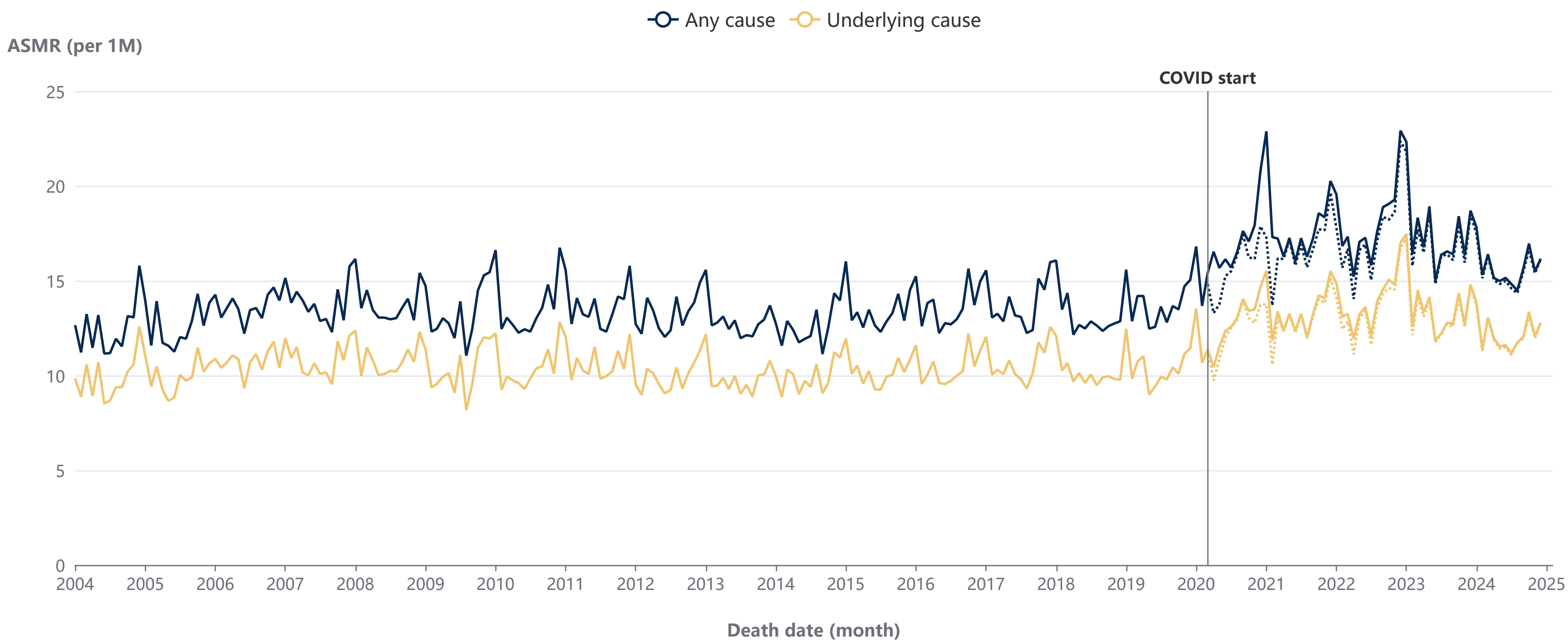

COVID start = March 2020

(B.1) Mean ASMR, and % step-change estimated using pre-COVID baseline of January 2004 to February 2020

	Mean ASMR (95% CI)			% step change (95% CI; P value)	
Category	Pre COVID	Post COVID (inc deaths)	Post COVID (exc deaths)	COVID deaths included	COVID deaths excluded
Any cause	13.36 (13.14 to 13.47)	17.24 (16.67 to 17.62)	16.50 (15.99 to 16.86)	27.0 (22.8 to 31.3; <0.001)	20.9 (16.7 to 25.3; <0.001)
Underlying cause	10.34 (10.16 to 10.43)	13.19 (12.77 to 13.48)	12.90 (12.48 to 13.19)	27.7 (23.3 to 32.3; <0.001)	24.6 (20.1 to 29.3; <0.001)

Pre-COVID = January 2004 to February 2020

Post-COVID = March 2020 to December 2024

(B.2) Mean ASMR, and % step-change estimated using pre-COVID baseline of January 2017 to February 2020

	Mean ASMR (95% CI)			% step change (95% CI; P value)	
Category	Pre COVID	Post COVID (inc deaths)	Post COVID (exc deaths)	COVID deaths included	COVID deaths excluded
Any cause	13.64 (13.20 to 13.98)	17.24 (16.67 to 17.62)	16.50 (15.99 to 16.86)	35.9 (28.1 to 44.1; <0.001)	21.8 (13.6 to 30.6; <0.001)
Underlying cause	10.50 (10.14 to 10.77)	13.19 (12.77 to 13.48)	12.90 (12.48 to 13.19)	30.8 (22.0 to 40.2; <0.001)	24.7 (15.4 to 34.7; <0.001)

Pre-COVID = January 2017 to February 2020

Post-COVID = March 2020 to December 2024
