# Supplementary material for: Temporal trends in alcohol-related liver disease mortality and prior inpatient care in England before, during and after the COVID-19 pandemic (2004–2024): a population-based observational study
Source: J Epidemiol Community Health. 2026 Aug 12;80(11):e225179. doi: 10.1136/jech-2025-225179 (PMC13691703; doi:10.1136/jech-2025-225179)
Supplement: Supplementary file 8 [file jech-80-11-s008.pdf]

**eTable 1. Distribution of deaths where alcohol-related liver disease (ICD-10 K70) was listed as an underlying cause in England between January 2004 and December 2024, by demographics, place of death, and (among out-of-hospital deaths) presence of any prior inpatient care. The table presents counts, with row proportions provided in parentheses.**

| | | Out-of-hospital | | | | |
|---|---|---|---|---|---|---|
| | In-hospital | Any discharge within 60d | Any discharge within 61d-1y | No discharge within 1y | | Total |
| All deaths | 67,018 (69) | 10,557 (10.9) | 8,642 (8.9) | 10,841 (11.2) | 30,040 (31) | 97,058 (100) |
| Age group | | | | | | |
| 25-39 | 4,853 (64.8) | 880 (11.8) | 795 (10.6) | 960 (12.8) | 2,635 (35.2) | 7,488 (100) |
| 40-54 | 24,805 (67.2) | 3,784 (10.2) | 3,789 (10.3) | 4,559 (12.3) | 12,132 (32.8) | 36,937 (100) |
| 55-69 | 29,247 (71) | 4,250 (10.3) | 3,275 (7.9) | 4,432 (10.8) | 11,957 (29) | 41,204 (100) |
| 70-84 | 8,113 (71) | 1,643 (14.4) | 783 (6.9) | 890 (7.8) | 3,316 (29) | 11,429 (100) |
| Sex | | | | | | |
| Female | 24,192 (72.6) | 3,219 (9.7) | 2,648 (7.9) | 3,282 (9.8) | 9,149 (27.4) | 33,341 (100) |
| Male | 42,826 (67.2) | 7,338 (11.5) | 5,994 (9.4) | 7,559 (11.9) | 20,891 (32.8) | 63,717 (100) |
| IMD quintile | | | | | | |
| 1 (Most deprived) | 23,645 (68.5) | 3,676 (10.7) | 3,186 (9.2) | 3,991 (11.6) | 10,853 (31.5) | 34,498 (100) |
| 2 | 15,144 (68.9) | 2,351 (10.7) | 1,990 (9.1) | 2,484 (11.3) | 6,825 (31.1) | 21,969 (100) |
| 3 | 11,538 (68.9) | 1,871 (11.2) | 1,443 (8.6) | 1,900 (11.3) | 5,214 (31.1) | 16,752 (100) |
| 4 | 9,388 (69.8) | 1,450 (10.8) | 1,201 (8.9) | 1,408 (10.5) | 4,059 (30.2) | 13,447 (100) |
| 5 (Least deprived) | 7,303 (70.3) | 1,209 (11.6) | 822 (7.9) | 1,058 (10.2) | 3,089 (29.7) | 10,392 (100) |
| Region | | | | | | |
| East Midlands | 6,232 (70.3) | 930 (10.5) | 772 (8.7) | 928 (10.5) | 2,630 (29.7) | 8,862 (100) |
| East of England | 5,371 (67.7) | 873 (11) | 726 (9.1) | 965 (12.2) | 2,564 (32.3) | 7,935 (100) |
| London | 6,748 (67.9) | 959 (9.7) | 873 (8.8) | 1,353 (13.6) | 3,185 (32.1) | 9,933 (100) |
| North East | 5,198 (72.3) | 765 (10.6) | 551 (7.7) | 678 (9.4) | 1,994 (27.7) | 7,192 (100) |
| North West | 13,248 (68.8) | 2,063 (10.7) | 1,796 (9.3) | 2,154 (11.2) | 6,013 (31.2) | 19,261 (100) |
| South East | 8,212 (67.9) | 1,435 (11.9) | 1,093 (9) | 1,354 (11.2) | 3,882 (32.1) | 12,094 (100) |
| South West | 6,217 (68.4) | 1,021 (11.2) | 913 (10) | 934 (10.3) | 2,868 (31.6) | 9,085 (100) |
| West Midlands | 8,106 (68.6) | 1,287 (10.9) | 1,034 (8.8) | 1,383 (11.7) | 3,704 (31.4) | 11,810 (100) |
| Yorkshire and the Humber | 7,686 (70.6) | 1,224 (11.2) | 884 (8.1) | 1,092 (10) | 3,200 (29.4) | 10,886 (100) |

IMD = Index of Multiple Deprivation
