# Supplementary material for: Temporal trends in alcohol-related liver disease mortality and prior inpatient care in England before, during and after the COVID-19 pandemic (2004–2024): a population-based observational study
Source: J Epidemiol Community Health. 2026 Aug 12;80(11):e225179. doi: 10.1136/jech-2025-225179 (PMC13691703; doi:10.1136/jech-2025-225179)
Supplement: Supplementary file 9 [file jech-80-11-s009.pdf]

eTable 2. Twenty most common diagnoses among people who died from alcohol-related liver disease (ICD-10 K70) in England, stratified by presence of hospital-recorded ARLD diagnosis in the year before death.

ARLD diagnosed in the last 12 months (n=74,586)			ARLD not diagnosed in the last 12 months (n=22,472)		
ICD-10 code (3-digit level)	Description	n (%)	ICD-10 code (3-digit level)	Description	n (%)
K70	Alcoholic liver disease	74,586 (100)		No discharge <1y	10,841 (48.2)
F10	Mental and behavioural disorders due to use of alcohol	56,556 (75.8)	F10	Mental and behavioural disorders due to use of alcohol	7,671 (34.1)
R18	Ascites	45,747 (61.3)	E87	Other disorders of fluid, electrolyte and acid-base balance	3,549 (15.8)
K76	Other diseases of liver	36,151 (48.5)	F17	Mental and behavioural disorders due to use of tobacco	3,140 (14)
E87	Other disorders of fluid, electrolyte and acid-base balance	34,676 (46.5)	N17	Acute renal failure	3,074 (13.7)
N17	Acute renal failure	33,888 (45.4)	K76	Other diseases of liver	2,942 (13.1)
Z86	Personal history of certain other diseases	28,087 (37.7)	I10	Essential (primary) hypertension	2,784 (12.4)
K92	Other diseases of digestive system	22,893 (30.7)	Z86	Personal history of certain other diseases	2,762 (12.3)
F17	Mental and behavioural disorders due to use of tobacco	22,859 (30.6)	R18	Ascites	2,452 (10.9)
J18	Pneumonia, organism unspecified	21,245 (28.5)	K70	Alcoholic liver disease	2,319 (10.3)
I10	Essential (primary) hypertension	20,894 (28)	F32	Depressive episode	2,265 (10.1)
Z51	Other medical care	19,789 (26.5)	J18	Pneumonia, organism unspecified	2,056 (9.1)
D64	Other anaemias	17,774 (23.8)	K92	Other diseases of digestive system	2,002 (8.9)
I98	Other disorders of circulatory system in diseases classified elsewhere	17,353 (23.3)	Z72	Problems related to lifestyle	1,518 (6.8)
F32	Depressive episode	16,373 (22)	K72	Hepatic failure, not elsewhere classified	1,499 (6.7)
K29	Gastritis and duodenitis	14,983 (20.1)	Z51	Other medical care	1,498 (6.7)
K72	Hepatic failure, not elsewhere classified	15,028 (20.1)	A41	Other sepsis	1,364 (6.1)
A41	Other sepsis	14,901 (20)	D64	Other anaemias	1,351 (6)
K31	Other diseases of stomach and duodenum	13,967 (18.7)	K29	Gastritis and duodenitis	1,356 (6)
J90	Pleural effusion, not elsewhere classified	13,773 (18.5)	Z50	Care involving use of rehabilitation procedures	1,341 (6)
