# Supplementary material for: Temporal trends in alcohol-related liver disease mortality and prior inpatient care in England before, during and after the COVID-19 pandemic (2004–2024): a population-based observational study
Source: J Epidemiol Community Health. 2026 Aug 12;80(11):e225179. doi: 10.1136/jech-2025-225179 (PMC13691703; doi:10.1136/jech-2025-225179)
Supplement: Supplementary file 10 [file jech-80-11-s010.pdf]

eText 1. Methods for deriving mid-year population estimates and addressing incomplete data from specified hospital trusts.

Population-based rates for England were calculated using mid-year population estimates from the Office for National Statistics (ONS). Two types of estimates were sourced: national rebased estimates (revised following the 2021 Census) covering 2004 –2023 (1), and Lower Layer Super Output Area (LSOA) estimates for 2004–2020 (2). The national rebased estimates included denominators by age group, sex, and for the total population. 2023 estimates were used for 2024 data.

English Indices of Deprivation (IMD), which assess relative deprivation across LSOAs (3), were used as the measure of area-level deprivation. IMD 2019, the most recent version, is based on 2011 Census boundaries, with the 2020 LSOA dataset being the final release to use those boundaries (1). IMD quintiles were mapped to LSOA estimates for 2004 to 2020: estimates from 2004–2016 were matched with IMD 2015, while those from 2017–2020 were matched with IMD 2019. The resulting annual IMD distribution was applied to the national rebased estimates by age and sex for 2004–2023 using the 2020 distribution for years 2021–2023. 2023 estimates were again used for 2024 denominators. The same method was applied at the Integrated Care Board (ICB) level, ICB classifications derived from the ONS correspondence table (4).

Frimley Health NHS Foundation Trust (RDU) was unable to provide complete HES records between June 2022 and March 2023 due to missing data reported by NHS England (5). Likewise, Shrewsbury and Telford Hospital NHS Trust (RXW) was unable to submit records from early July 2024 (6). These omissions affected national counts. To prevent underestimation of ARLD rates, all records from two ICBs were excluded: NHS Shropshire, Telford and Wrekin Integrated Care Board, and NHS Frimley Integrated Care Board. In a typical year with complete data submission, deaths occurring among residents of these two ICBs combined accounted for approximately 1.9% of all ARLD deaths nationally. Across the full study period, a total of 1,909 (1.9%) deaths were excluded as a result. The corresponding baseline populations for these ICBs were also removed from the population denominators.

References

1. Office for National Statistics (ONS). nomis (official census and labour market statistics). 2025 [cited 2025 Jun 25]. Population estimates - local authority based by single year of age. Available from: https://www.nomisweb.co.uk/datasets/pestsyoala

2. Office for National Statistics (ONS). Office for National Statistics. 2025 [cited 2025 Jun 25]. Lower layer Super Output Area population estimates (supporting information). Available from: https://www.ons.gov.uk/peoplepopulationandcommunity/populationandmigration/populationestimates/datasets/lowersuperoutputareamidyearpopulationestimates

3. Department for Levelling Up, Housing and Communities. GOV.UK. 2025 [cited 2025 May 26]. English indices of deprivation. Available from: https://www.gov.uk/government/collections/english-indices-of-deprivation

4. LSOA (2011) to Sub Integrated Care Board Locations (July 2022) Lookup in EN [Internet]. [cited 2025 Jun 25]. Available from: https://geoportal.statistics.gov.uk/datasets/ons::lsoa-2011-to-sub-integrated-care-board-locations-july-2022-lookup-in-en/about

5. NHS England. Digital. 2023 [cited 2026 Jan 16]. Hospital Admitted Patient Care Activity, 2022-23. Available from: https://digital.nhs.uk/data-and-information/publications/statistical/hospital-admitted-patient-care-activity/2022-23/about-this-publication

6. NHS England. Digital. 2025 [cited 2025 May 26]. Hospital Admitted Patient Care Activity. Available from: https://digital.nhs.uk/data-and-information/publications/statistical/hospital-admitted-patient-care-activity
